# 1-Methyl-2-(4-methyl­phen­yl)-4-morpholinopyridazine-3,6(1*H*,2*H*)-dione

**DOI:** 10.1107/S1600536808000111

**Published:** 2008-01-11

**Authors:** Andrzej Gzella, Ewa Melzer, Michał S. Kaczmarek, Zenon Woźniak

**Affiliations:** a Department of Organic Chemistry, Poznan University of Medical Sciences, ul. Grunwaldzka 6, 60-780 Poznań, Poland; b Optics Division, Faculty of Physics, A. Mickiewicz University, ul. Umultowska 85, 61-614 Poznań, Poland

## Abstract

The structure analysis of the title compound, C_16_H_19_N_3_O_3_, has been undertaken in order to facilitate the inter­pretation of ^1^H and ^13^C NMR data and to determine the position of the morpholine residue in this nucleophilic substitution product. The main result is that the morpholine group, with a chair conformation, is connected at the 4-position of the pyridazine ring. The benzene and pyridazine rings make a dihedral angle of 62.17 (5)°. Mol­ecules are linked into a two-dimensional network by non-classical C—H⋯O hydrogen bonds, in which O atoms serve as double or triple acceptors.

## Related literature

For related literature, see: Allen *et al.* (1987 ▶); Bałoniak & Melzer (1979 ▶); Katrusiak *et al.* (2002 ▶).
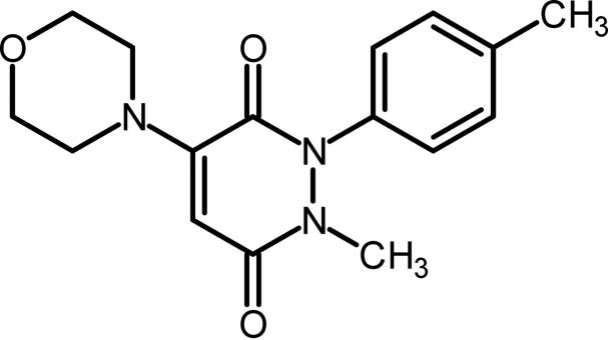


## Experimental

### 

#### Crystal data

C_16_H_19_N_3_O_3_
*M* *_r_* = 301.34
Monoclinic, 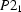

*a* = 5.6246 (6) Å
*b* = 8.8923 (6) Å
*c* = 15.0842 (10) Å
β = 99.530 (7)°
*V* = 744.03 (11) Å^3^
*Z* = 2
Cu *K*α radiation
μ = 0.78 mm^−1^
*T* = 293 (2) K
0.38 × 0.35 × 0.30 mm

#### Data collection

Kuma Diffraction KM-4 diffractometer
Absorption correction: ψ scan (North *et al.*, 1968 ▶) *T*_min_ = 0.716, *T*_max_ = 0.794
2796 measured reflections
2705 independent reflections
2646 reflections with *I* > 2σ(*I*)
*R*_int_ = 0.025
2 standard reflections every 100 reflections intensity decay: <2%

#### Refinement

*R*[*F* ^2^ > 2σ(*F* ^2^)] = 0.029
*wR*(*F* ^2^) = 0.082
*S* = 1.06
2705 reflections
202 parameters
1 restraint
H-atom parameters constrained
Δρ_max_ = 0.15 e Å^−3^
Δρ_min_ = −0.13 e Å^−3^
Absolute structure: Flack (1983 ▶); 1249 Friedel pairs
Flack parameter: 0.07 (16)

### 

Data collection: *KM-4 Software* (Kuma, 1996 ▶); cell refinement: *KM-4 Software*; data reduction: *KM-4 Software*; program(s) used to solve structure: *SHELXS97* (Sheldrick, 2008 ▶); program(s) used to refine structure: *SHELXL97* (Sheldrick, 2008 ▶); molecular graphics: *ORTEP-3 for Windows* (Farrugia, 1997 ▶); software used to prepare material for publication: *WinGX* (Farrugia, 1999 ▶).

## Supplementary Material

Crystal structure: contains datablocks I, global. DOI: 10.1107/S1600536808000111/cf2177sup1.cif

Structure factors: contains datablocks I. DOI: 10.1107/S1600536808000111/cf2177Isup2.hkl

Additional supplementary materials:  crystallographic information; 3D view; checkCIF report

## Figures and Tables

**Table 1:** Hydrogen-bond geometry (Å, °)

*D*—H⋯*A*	*D*—H	H⋯*A*	*D*⋯*A*	*D*—H⋯*A*
C17—H17*A*⋯O15	0.97	2.21	2.8636 (18)	124
C10—H10⋯O15^i^	0.93	2.53	3.3508 (19)	148
C14—H14*C*⋯O15^i^	0.96	2.53	3.419 (2)	155
C5—H5⋯O19^ii^	0.93	2.49	3.4124 (19)	174
C21—H21*B*⋯O19^ii^	0.97	2.52	3.3036 (18)	137

Symmetry codes: (i) 
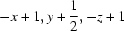
; (ii) 
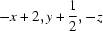
.

**Fig. 1.**
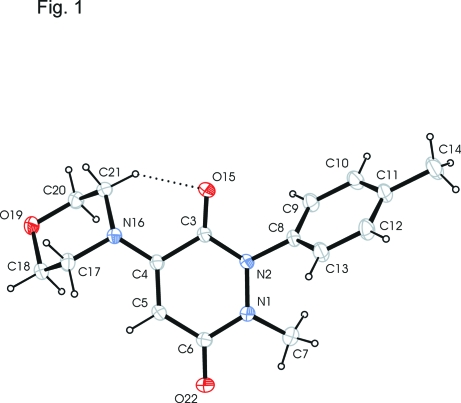
The molecular structure of (I), showing the atomic labelling scheme. Non-H atoms are drawn as 30% probability displacement ellipsoids.

**Fig. 2.**
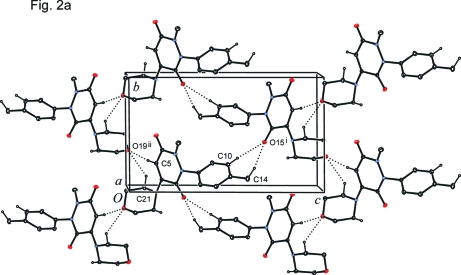
Molecular packing and hydrogen bonds (dotted lines); symmetry codes: (i) 1 - x, 1/2 + y, 1 - z, (ii) 2 - x, 1/2 + y, -z. H atoms not involved in hydrogen bonds have been omitted for clarity.

**Fig. 3.**
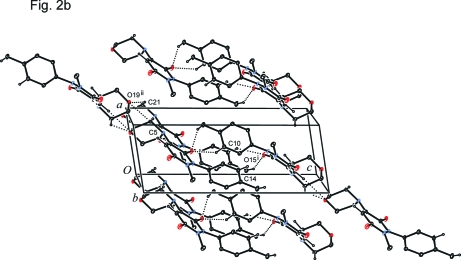
Molecular packing and hydrogen bonds (dotted lines); symmetry codes: (i) 1 - x, 1/2 + y, 1 - z, (ii) 2 - x, 1/2 + y, -z. H atoms not involved in hydrogen bonds have been omitted for clarity.

## supplementary crystallographic information

### Comment

Treatment of 4-bromo-1-methyl-2-(4-methylphenyl)-3,6-pyridazinedione with morpholine in anhydrous ethanol gives a mixture of *ipso* and *cine* substitution products; one of them, labelled as (I), was found as a precipitate (Bałoniak & Melzer, 1979). The crystal structure determination of (I) was carried out in order to facilitate the interpretation of ^1^H and ^13^C NMR data, to determine the position of the morpholine residue on the pyridazinedione ring, and to study the nature of the hydrogen-bond formation in the crystalline state.

The X-ray analysis revealed the molecular structure of (I) and its conformation and distortions induced in the pyridazine ring by substituents.

The geometry of the molecule of (I) is illustrated in Fig. 1. The pyridazine ring is nearly planar with an r.m.s. deviation of 0.0211 Å. The methyl, *p*-methylphenyl and morpholine substituents are connected at N1, N2 and C4, respectively. The mean plane of the benzene ring is oriented at an angle of 62.17 (5)° to the mean plane of the pyridazine ring. The C4—C5 bond, 1.3500 (18) Å, belonging to the latter ring, is a double bond.

The ring bonds are conjugated, and the formally single bond C5—C6 is shorter by about 14 and the bond C3—C4 is longer by about 13σ than the normal (C?)C*sp*^2^—C*sp*^2^(?O) single bond [1.465 (1) Å; Allen *et al.*, 1987]. The elongation of the latter is a result of the presence of the morpholine residue at C4. The last two observations are consistent with that reported for 2-methyl-4-morpholino-1-phenyl-3,6-pyridazinedione (Katrusiak *et al.*, 2002).

The C3—N1 and C6—N1 distances are similar [1.3700 (18) and 1.3686 (17) Å, respectively] and are somewhat larger than a normal C—N tertiary amide distance [1.346 (5) Å; Allen *et al.*, 1987]. The sums of valency angles around N1 and N2 atoms are 356.5 and 357.4°. Atom C7 of the methyl group has a mutual orientation of synperiplanar and synclinal with respect to the atom C8 of the benzene ring [torsion angle C7—N1—N2—C8 = -37.79 (18)°].

The molecules in the crystal structure of (I) are linked *via* non-classical C—H···O hydrogen bonds (Table 1), forming a two-dimensional hydrogen-bond network parallel to the (101) plane (Figs. 2 and 3).

### Experimental

Compound (I) was synthesized according to a literature procedure of Bałoniak & Melzer (1979). Crystals suitable for single-crystal X-ray diffraction analysis were grown from ethanol by slow evaporation.

### Refinement

All H atoms were placed in geometrically calculated positions and were refined with a riding model with C—H = 0.93–0.97 Å and with *U*_iso_(H) = 1.2*U*_eq_(C) or 1.5*U*_eq_(C) for methyl H. The methyl groups were refined as rigid groups, allowed to rotate. The crystal polarity of (I) was established by refinement of the Flack (1983) parameter. The relatively large s.u. of the Flack parameter is due to the small contribution of atoms with measurable anomalous dispersion effects.

### Crystal data

**Table:**

C_16_H_19_N_3_O_3_	*F*(000) = 320
*M**_r_* = 301.34	*D*_x_ = 1.345 Mg m^−^^3^
Monoclinic, *P*2_1_	Melting point = 475–476 K
Hall symbol: P 2yb	Cu *K*α radiation, λ = 1.54178 Å
*a* = 5.6246 (6) Å	Cell parameters from 53 reflections
*b* = 8.8923 (6) Å	θ = 14.8–30.5°
*c* = 15.0842 (10) Å	µ = 0.78 mm^−^^1^
β = 99.530 (7)°	*T* = 293 K
*V* = 744.03 (11) Å^3^	Block, colourless
*Z* = 2	0.38 × 0.35 × 0.30 mm

### Data collection

**Table:**

Kuma Diffraction KM-4 diffractometer	2646 reflections with *I* > 2σ(*I*)
Radiation source: fine-focus sealed tube	*R*_int_ = 0.025
graphite	θ_max_ = 70.1°, θ_min_ = 3.0°
ω–2θ scans	*h* = −6→6
Absorption correction: ψ scan (North *et al.*, 1968)	*k* = −10→10
*T*_min_ = 0.716, *T*_max_ = 0.794	*l* = 0→18
2796 measured reflections	2 standard reflections every 100 reflections
2705 independent reflections	intensity decay: <2%

### Refinement

**Table:**

Refinement on *F*^2^	Hydrogen site location: inferred from neighbouring sites
Least-squares matrix: full	H-atom parameters constrained
*R*[*F*^2^ > 2σ(*F*^2^)] = 0.029	*w* = 1/[σ^2^(*F*_o_^2^) + (0.0538*P*)^2^ + 0.0723*P*] where *P* = (*F*_o_^2^ + 2*F*_c_^2^)/3
*wR*(*F*^2^) = 0.082	(Δ/σ)_max_ = 0.001
*S* = 1.07	Δρ_max_ = 0.15 e Å^−^^3^
2705 reflections	Δρ_min_ = −0.13 e Å^−^^3^
202 parameters	Extinction correction: *SHELXL97*, Fc^*^=kFc[1+0.001xFc^2^λ^3^/sin(2θ)]^-1/4^
1 restraint	Extinction coefficient: 0.0345 (18)
Primary atom site location: structure-invariant direct methods	Absolute structure: Flack (1983); 1249 Fiedel pairs
Secondary atom site location: difference Fourier map	Flack parameter: 0.07 (16)

### Special details

**Table:**

Geometry. All e.s.d.'s (except the e.s.d. in the dihedral angle between two l.s. planes) are estimated using the full covariance matrix. The cell e.s.d.'s are taken into account individually in the estimation of e.s.d.'s in distances, angles and torsion angles; correlations between e.s.d.'s in cell parameters are only used when they are defined by crystal symmetry. An approximate (isotropic) treatment of cell e.s.d.'s is used for estimating e.s.d.'s involving l.s. planes.
Refinement. Refinement of *F*^2^ against all reflections. The weighted *R*-factor *wR* and goodness of fit *S* are based on *F*^2^, conventional *R*-factors *R* are based on *F*, with *F* set to zero for negative *F*^2^. The threshold expression of *F*^2^ > σ(*F*^2^) is used only for calculating *R*-factors(gt) *etc*. and is not relevant to the choice of reflections for refinement. *R*-factors based on *F*^2^ are statistically about twice as large as those based on *F*, and *R*- factors based on ALL data will be even larger.

### Fractional atomic coordinates and isotropic or equivalent isotropic displacement parameters (Å^2^)

**Table:**

	*x*	*y*	*z*	*U*_iso_*/*U*_eq_	
N1	0.4950 (2)	0.30562 (12)	0.24834 (8)	0.0381 (3)	
N2	0.4743 (2)	0.15882 (12)	0.28125 (7)	0.0339 (3)	
C3	0.5939 (3)	0.03738 (15)	0.25416 (8)	0.0325 (3)	
C4	0.7375 (2)	0.06485 (14)	0.18118 (8)	0.0300 (3)	
C5	0.7609 (3)	0.20788 (15)	0.15387 (9)	0.0359 (3)	
H5	0.8658	0.2264	0.1135	0.043*	
C6	0.6334 (3)	0.33293 (15)	0.18353 (9)	0.0369 (3)	
C7	0.3067 (3)	0.41229 (19)	0.25978 (12)	0.0516 (4)	
H7A	0.3504	0.4642	0.3159	0.077*	
H7B	0.1577	0.3595	0.2596	0.077*	
H7C	0.2876	0.4837	0.2114	0.077*	
C8	0.3819 (2)	0.14755 (15)	0.36480 (8)	0.0326 (3)	
C9	0.4878 (3)	0.22780 (18)	0.43950 (9)	0.0426 (3)	
H9	0.6209	0.2886	0.4367	0.051*	
C10	0.3942 (3)	0.2170 (2)	0.51839 (10)	0.0450 (4)	
H10	0.4645	0.2717	0.5684	0.054*	
C11	0.1978 (3)	0.12600 (18)	0.52421 (9)	0.0408 (3)	
C12	0.0964 (3)	0.04506 (18)	0.44820 (10)	0.0425 (3)	
H12	−0.0346	−0.0175	0.4510	0.051*	
C13	0.1868 (3)	0.05591 (17)	0.36881 (9)	0.0381 (3)	
H13	0.1165	0.0018	0.3185	0.046*	
C14	0.0953 (4)	0.1141 (2)	0.61008 (11)	0.0581 (5)	
H14A	0.1592	0.0263	0.6427	0.087*	
H14B	−0.0771	0.1064	0.5961	0.087*	
H14C	0.1385	0.2020	0.6461	0.087*	
O15	0.5897 (2)	−0.08309 (11)	0.29241 (7)	0.0475 (3)	
N16	0.8655 (2)	−0.05448 (13)	0.15642 (7)	0.0339 (3)	
C17	0.7617 (3)	−0.20541 (15)	0.13920 (9)	0.0369 (3)	
H17A	0.6324	−0.2196	0.1737	0.044*	
H17B	0.8843	−0.2807	0.1582	0.044*	
C18	0.6651 (3)	−0.22466 (16)	0.04052 (10)	0.0407 (3)	
H18A	0.6047	−0.3264	0.0299	0.049*	
H18B	0.5311	−0.1561	0.0234	0.049*	
O19	0.8440 (2)	−0.19668 (12)	−0.01424 (7)	0.0444 (3)	
C20	0.9381 (3)	−0.04743 (17)	0.00089 (9)	0.0404 (3)	
H20A	0.8098	0.0252	−0.0157	0.049*	
H20B	1.0604	−0.0298	−0.0363	0.049*	
C21	1.0461 (3)	−0.02754 (16)	0.09851 (9)	0.0386 (3)	
H21A	1.1792	−0.0972	0.1142	0.046*	
H21B	1.1088	0.0738	0.1082	0.046*	
O22	0.6396 (2)	0.46036 (12)	0.15195 (8)	0.0522 (3)	

### Atomic displacement parameters (Å^2^)

**Table:**

	*U*^11^	*U*^22^	*U*^33^	*U*^12^	*U*^13^	*U*^23^
N1	0.0510 (7)	0.0290 (6)	0.0389 (6)	0.0038 (5)	0.0206 (5)	0.0022 (5)
N2	0.0453 (7)	0.0291 (5)	0.0311 (5)	−0.0018 (5)	0.0176 (5)	0.0000 (4)
C3	0.0425 (7)	0.0313 (6)	0.0260 (5)	−0.0011 (5)	0.0122 (5)	−0.0013 (5)
C4	0.0367 (7)	0.0308 (6)	0.0236 (5)	−0.0012 (5)	0.0083 (5)	−0.0018 (4)
C5	0.0458 (8)	0.0327 (7)	0.0332 (6)	−0.0023 (6)	0.0181 (5)	0.0008 (5)
C6	0.0489 (8)	0.0287 (7)	0.0362 (7)	−0.0036 (6)	0.0157 (6)	−0.0004 (5)
C7	0.0578 (10)	0.0419 (8)	0.0617 (9)	0.0102 (7)	0.0289 (8)	0.0014 (7)
C8	0.0383 (7)	0.0353 (7)	0.0268 (6)	−0.0004 (5)	0.0131 (5)	−0.0021 (5)
C9	0.0449 (9)	0.0472 (8)	0.0393 (7)	−0.0128 (6)	0.0174 (6)	−0.0094 (6)
C10	0.0507 (9)	0.0540 (8)	0.0324 (6)	−0.0070 (7)	0.0131 (6)	−0.0121 (6)
C11	0.0462 (8)	0.0462 (8)	0.0332 (7)	0.0025 (6)	0.0163 (6)	0.0023 (6)
C12	0.0399 (8)	0.0488 (8)	0.0418 (7)	−0.0085 (6)	0.0153 (6)	0.0005 (6)
C13	0.0385 (7)	0.0445 (8)	0.0319 (6)	−0.0063 (6)	0.0082 (5)	−0.0042 (5)
C14	0.0687 (11)	0.0728 (12)	0.0394 (8)	−0.0065 (9)	0.0278 (7)	0.0017 (8)
O15	0.0746 (7)	0.0334 (5)	0.0418 (5)	0.0051 (5)	0.0307 (5)	0.0098 (4)
N16	0.0431 (6)	0.0301 (6)	0.0311 (5)	−0.0007 (5)	0.0140 (4)	−0.0022 (4)
C17	0.0511 (8)	0.0264 (6)	0.0363 (7)	−0.0004 (6)	0.0165 (6)	−0.0002 (5)
C18	0.0489 (8)	0.0350 (7)	0.0415 (7)	−0.0048 (6)	0.0173 (6)	−0.0072 (6)
O19	0.0602 (7)	0.0384 (6)	0.0397 (5)	−0.0032 (5)	0.0237 (5)	−0.0100 (4)
C20	0.0514 (8)	0.0364 (7)	0.0389 (7)	−0.0003 (6)	0.0234 (6)	−0.0009 (6)
C21	0.0373 (7)	0.0382 (7)	0.0435 (7)	0.0015 (6)	0.0163 (5)	−0.0039 (6)
O22	0.0788 (8)	0.0287 (5)	0.0565 (6)	0.0005 (5)	0.0326 (6)	0.0058 (5)

### Geometric parameters (Å, °)

**Table:**

N1—C6	1.3686 (17)	C11—C14	1.5062 (18)
N1—N2	1.4083 (15)	C12—C13	1.3796 (18)
N1—C7	1.4530 (19)	C12—H12	0.930
N2—C3	1.3700 (18)	C13—H13	0.930
N2—C8	1.4440 (15)	C14—H14A	0.960
C3—O15	1.2188 (17)	C14—H14B	0.960
C3—C4	1.4897 (16)	C14—H14C	0.960
C4—C5	1.3500 (18)	N16—C21	1.4648 (15)
C4—N16	1.3685 (17)	N16—C17	1.4696 (17)
C5—C6	1.4344 (19)	C17—C18	1.507 (2)
C5—H5	0.930	C17—H17A	0.970
C6—O22	1.2319 (18)	C17—H17B	0.970
C7—H7A	0.960	C18—O19	1.4256 (16)
C7—H7B	0.960	C18—H18A	0.970
C7—H7C	0.960	C18—H18B	0.970
C8—C13	1.376 (2)	O19—C20	1.4330 (18)
C8—C9	1.3826 (19)	C20—C21	1.507 (2)
C9—C10	1.3819 (18)	C20—H20A	0.970
C9—H9	0.930	C20—H20B	0.970
C10—C11	1.384 (2)	C21—H21A	0.970
C10—H10	0.930	C21—H21B	0.970
C11—C12	1.393 (2)		
			
C6—N1—N2	120.42 (11)	C11—C12—H12	119.4
C6—N1—C7	118.75 (12)	C8—C13—C12	119.38 (12)
N2—N1—C7	117.31 (11)	C8—C13—H13	120.3
C3—N2—N1	123.52 (11)	C12—C13—H13	120.3
C3—N2—C8	118.10 (11)	C11—C14—H14A	109.5
N1—N2—C8	115.76 (10)	C11—C14—H14B	109.5
O15—C3—N2	120.18 (11)	H14A—C14—H14B	109.5
O15—C3—C4	123.32 (12)	C11—C14—H14C	109.5
N2—C3—C4	116.39 (11)	H14A—C14—H14C	109.5
C5—C4—N16	124.41 (12)	H14B—C14—H14C	109.5
C5—C4—C3	118.17 (11)	C4—N16—C21	118.94 (11)
N16—C4—C3	116.66 (11)	C4—N16—C17	123.00 (11)
C4—C5—C6	123.79 (12)	C21—N16—C17	109.79 (10)
C4—C5—H5	118.1	N16—C17—C18	110.23 (11)
C6—C5—H5	118.1	N16—C17—H17A	109.6
O22—C6—N1	119.68 (12)	C18—C17—H17A	109.6
O22—C6—C5	123.02 (12)	N16—C17—H17B	109.6
N1—C6—C5	117.28 (11)	C18—C17—H17B	109.6
N1—C7—H7A	109.5	H17A—C17—H17B	108.1
N1—C7—H7B	109.5	O19—C18—C17	112.26 (12)
H7A—C7—H7B	109.5	O19—C18—H18A	109.2
N1—C7—H7C	109.5	C17—C18—H18A	109.2
H7A—C7—H7C	109.5	O19—C18—H18B	109.2
H7B—C7—H7C	109.5	C17—C18—H18B	109.2
C13—C8—C9	120.59 (12)	H18A—C18—H18B	107.9
C13—C8—N2	118.96 (11)	C18—O19—C20	110.30 (10)
C9—C8—N2	120.45 (12)	O19—C20—C21	110.04 (12)
C10—C9—C8	119.45 (14)	O19—C20—H20A	109.7
C10—C9—H9	120.3	C21—C20—H20A	109.7
C8—C9—H9	120.3	O19—C20—H20B	109.7
C9—C10—C11	121.15 (14)	C21—C20—H20B	109.7
C9—C10—H10	119.4	H20A—C20—H20B	108.2
C11—C10—H10	119.4	N16—C21—C20	110.87 (11)
C10—C11—C12	118.17 (12)	N16—C21—H21A	109.5
C10—C11—C14	121.26 (14)	C20—C21—H21A	109.5
C12—C11—C14	120.57 (15)	N16—C21—H21B	109.5
C13—C12—C11	121.25 (13)	C20—C21—H21B	109.5
C13—C12—H12	119.4	H21A—C21—H21B	108.1
			
C6—N1—N2—C3	2.4 (2)	N1—N2—C8—C9	−53.57 (18)
C7—N1—N2—C3	160.94 (14)	C13—C8—C9—C10	−0.8 (2)
C6—N1—N2—C8	163.62 (12)	N2—C8—C9—C10	179.08 (14)
C7—N1—N2—C8	−37.79 (18)	C8—C9—C10—C11	0.7 (3)
N1—N2—C3—O15	171.72 (13)	C9—C10—C11—C12	0.1 (3)
C8—N2—C3—O15	10.9 (2)	C9—C10—C11—C14	−179.93 (16)
N1—N2—C3—C4	−4.50 (19)	C10—C11—C12—C13	−0.7 (2)
C8—N2—C3—C4	−165.37 (11)	C14—C11—C12—C13	179.35 (15)
O15—C3—C4—C5	−169.22 (15)	C9—C8—C13—C12	0.3 (2)
N2—C3—C4—C5	6.88 (19)	N2—C8—C13—C12	−179.65 (13)
O15—C3—C4—N16	1.3 (2)	C11—C12—C13—C8	0.5 (2)
N2—C3—C4—N16	177.35 (12)	C5—C4—N16—C21	1.9 (2)
N16—C4—C5—C6	−177.11 (13)	C3—C4—N16—C21	−167.96 (11)
C3—C4—C5—C6	−7.4 (2)	C5—C4—N16—C17	−143.29 (14)
N2—N1—C6—O22	176.13 (14)	C3—C4—N16—C17	46.90 (17)
C7—N1—C6—O22	17.8 (2)	C4—N16—C17—C18	93.59 (14)
N2—N1—C6—C5	−2.2 (2)	C21—N16—C17—C18	−54.29 (15)
C7—N1—C6—C5	−160.51 (14)	N16—C17—C18—O19	55.96 (15)
C4—C5—C6—O22	−173.17 (15)	C17—C18—O19—C20	−58.23 (16)
C4—C5—C6—N1	5.1 (2)	C18—O19—C20—C21	58.88 (15)
C3—N2—C8—C13	−71.32 (17)	C4—N16—C21—C20	−92.81 (14)
N1—N2—C8—C13	126.34 (14)	C17—N16—C21—C20	56.56 (15)
C3—N2—C8—C9	108.77 (16)	O19—C20—C21—N16	−58.94 (14)

### Hydrogen-bond geometry (Å, °)

**Table:**

*D*—H···*A*	*D*—H	H···*A*	*D*···*A*	*D*—H···*A*
C17—H17A···O15	0.97	2.21	2.8636 (18)	124
C10—H10···O15^i^	0.93	2.53	3.3508 (19)	148
C14—H14C···O15^i^	0.96	2.53	3.419 (2)	155
C5—H5···O19^ii^	0.93	2.49	3.4124 (19)	174
C21—H21B···O19^ii^	0.97	2.52	3.3036 (18)	137

Symmetry codes: (i) −*x*+1, *y*+1/2, −*z*+1; (ii) −*x*+2, *y*+1/2, −*z*.
